# Spectral-domain optical coherence tomography characteristics of cystic retinal tuft

**DOI:** 10.1186/s12886-022-02636-z

**Published:** 2022-10-28

**Authors:** Haidong Li, Lifeng Chen, Meng’ai Wu, Bin Zheng

**Affiliations:** Department of Retina Center, Eye Hospital of Wenzhou Medical University, #618 Fengqi East Road, Shangcheng District, Zhejiang Province 310020 Hangzhou City, China

**Keywords:** Cystic retinal tuft, Spectral-domain optical coherence tomography

## Abstract

**Objective:**

To investigate the characteristics of cystic retinal tufts (CRTs) with 55° widefield spectral-domain optical coherence tomography.

**Methods:**

This was a retrospective study. All subjects underwent a complete ocular examination, ultra-widefield (UWF) pseudocolor fundus photography and Spectral domain optical coherence tomography (SD-OCT) with a 55° widefield lens. The SD-OCT characteristics were analyzed in subjects with CRT.

**Results:**

Twenty-six eyes of 25 subjects were scanned and 29 CRTs were analyzed for SD-OCT characteristics. On SD-OCT images, the CRTs exhibited hyperreflective irregular elevated lesions with internal hyporeflective cystoid cavities. Normal layers of the neuroepithelium could not be distinguished. The mean diameter of CRTs was 1022 microns (range, 117–3711 microns; standard deviation, 815 microns). There was vitreoretinal traction at the apex of CRTs. Among them, retinal tears in 24.14% (7/29), suspected retinal tears in 27.59% (8/29), and shallow neuroepithelium detachment in 31.03% (9/29).

**Conclusions:**

The widefield SD-OCT imaging can provide detailed cross-sectional anatomic information of CRT and may guide clinical treatment.

## Introduction

A Cystic retinal tuft (CRT) is a developmental vitreoretinal abnormality in which retina is stretched by the vitreous and persistent vitreous traction may lead to retinal tears and detachment [1]. CRTs can be found in any part of the retina, but frequently in the peripheral retina lying posterior to the vitreous base. Histopathological analysis demonstrates a dome-shaped area with internal microcysts, glial cell proliferation, outer retinal degeneration, and photoreceptor loss [2], while one disadvantage of histopathology is that it is invasive and cannot be used for disease detection in large populations. For decades, slit-lamp biomicroscope and fundus photography were the primary diagnostic techniques for evaluating CRT in vivo, but today, modern high-resolution retinal imaging technology has revolutionized the precision of clinical diagnosis and treatment decisions. Spectral-domain optical coherence tomography (SD-OCT) allows us to evaluate retinal tissue layer by layer noninvasively and to guide clinical decision-making effectively [3, 4]. Despite SD-OCT has become an essential imaging technology for the diagnosis and treatment of macular diseases, it is rarely used to examine the peripheral retina. SD-OCT imaging of the retinal periphery is feasible and provides detailed anatomic information of lesions in the peripheral retina [5]. The purpose of this study was to obtain cross-sectional structural images of CRT by using 55° widefield SD-OCT and to aid in the diagnosis and management of this condition.

## Subjects and methods

This was a retrospective observational study. The procedures used in this research adhered to the tenets of the Declaration of Helsinki, and Institutional Review Board approval for this study was obtained from the Ethics Committee of Eye Hospital of Wenzhou Medical University. From January 2019 to December 2020, a total of 26 eyes of 25 subjects (11 males and 14 females) with previous clinical diagnosis of CRT were examined by ultra-widefield (UWF) pseudocolor imaging and 55° widefield SD-OCT imaging. Subjects ranged in age from 18 to 68 years. Each eye was dilated for 20 min before imaging examination. The UWF pseudocolor image was obtained by the Optos Tx-200 machine (Optos, Marlborough, MA). The SD-OCT image of CRT was obtained by the SD-OCT machine (Heidelberg engineering, Germany), which is equipped with a 55° widefield lens and a corresponding software (Heidelberg Eye Explorer 1.10.2.0). During SD-OCT scanning, a single line scan was performed in a vertical, horizontal, or oblique direction according to the position and nature of the CRT, and the patient's gaze was oriented in the desired direction, with the head slightly turned to the CRT area.

## Results

**Fig. 1:**
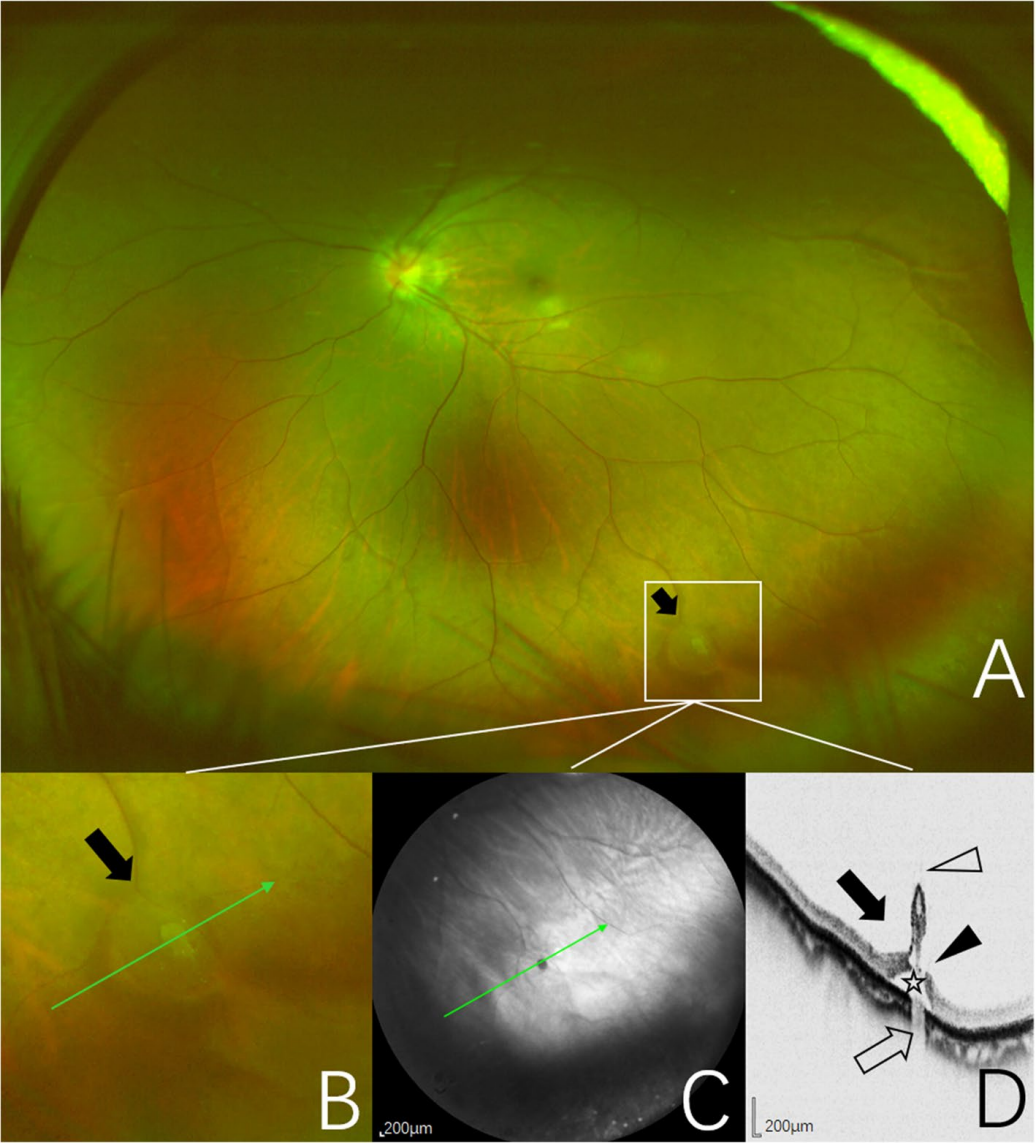
Images obtained from the left eye of a 21-year-old woman. **A** UWF pseudocolor image of one CRT (black arrow), outlined by the inset. **B** Pseudocolor image of magnification of CRT area outlined by the inset showing a focal, circular, cotton ball-like elevated lesion (black arrow). The green arrow indicates the section captured by the SD-OCT in (**C**, **D**). **C** Near-infrared scanning laser ophthalmoscopy image with green arrow show the position of SD-OCT cross-section. **D** SD-OCT image of the CRT showing a hyperreflective ovoid apical region containing hyporeflective cavities, which likely represents cystic regions (black arrow). At the apex, blurred hyperreflectivity represents vitreoretinal adhesion appearing to exert focal traction (open arrow). Shallow neuroepithelium detachment (open asterisk), a full-thickness retinal tear (black arrowhead). Decreased choroidal reflectivity at the level of the retinal elevation (open arrowhead)

**Fig. 2:**
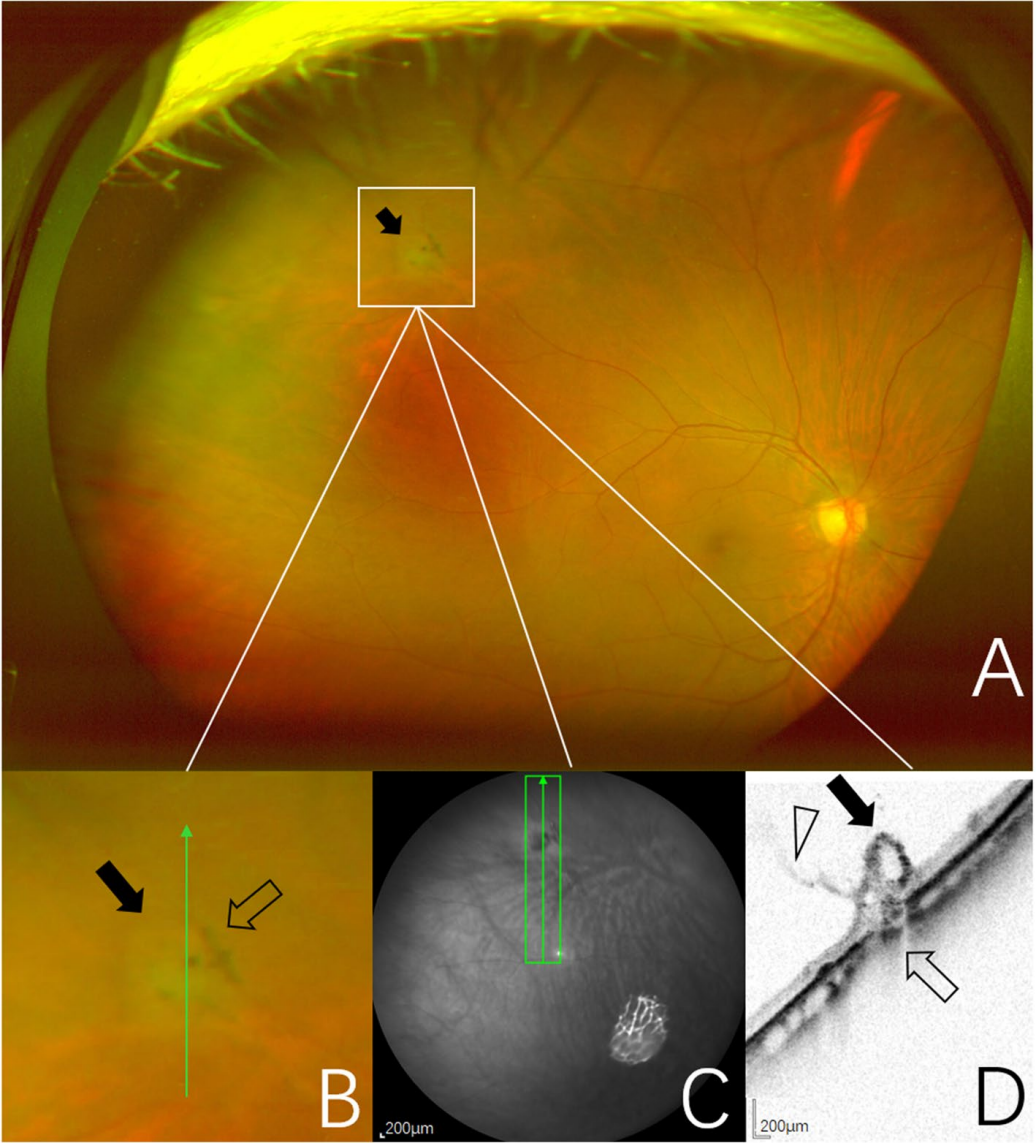
Images obtained from the right eye of a 63-year-old woman. **A** UWF pseudocolor image of one CRT (black arrow), outlined by the inset. **B** Pseudocolor image of magnification of CRT area outlined by the inset showing a focal, circular, cotton ball-like elevated lesion (black arrow). Pigment clumps are seen at the edges of the area of CRT (open arrow). The green arrow indicates the section captured by the SD-OCT in (**C**, **D**). **C** Near-infrared scanning laser ophthalmoscopy image with green arrow show the position of SD-OCT cross-section. **D** SD-OCT image of the CRT showing an irregular retinal region with hyporeflective cavities (black arrow). The vitreous is adherent (open arrowhead) and it appears to exert focal traction. The normally hyperreflective RPE layer is focally absent (open arrow)

**Fig. 3:**
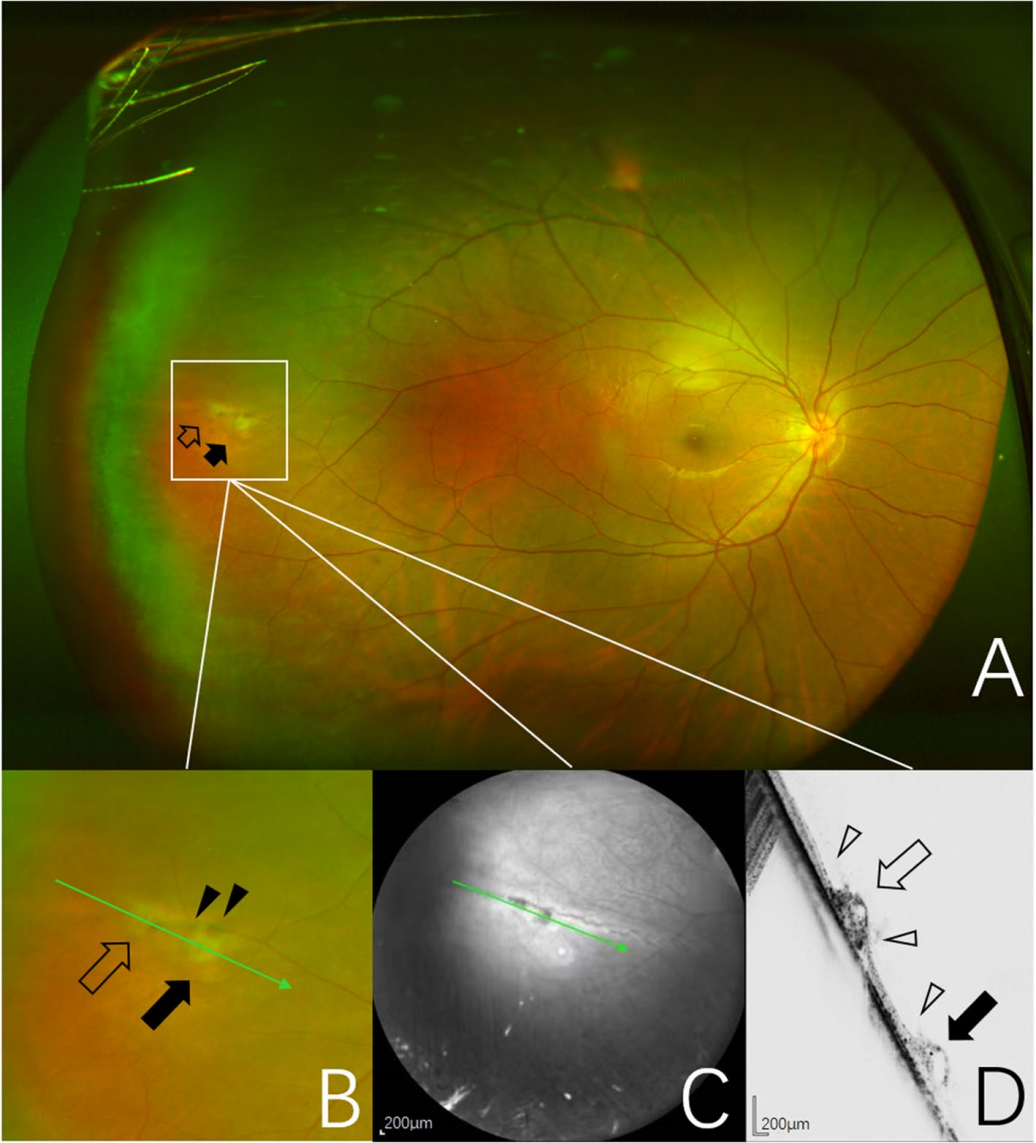
Images obtained from the right eye of a 20-year-old man. **A** UWF pseudocolor image of one CRTs (open and black arrows), outlined by the inset. **B** Pseudocolor image of magnification of CRTs area outlined by the inset showing focal gray lesions (open and black arrows) with pigmentation at the edge (black arrowheads). The green arrow indicates the section captured by the SD-OCT in (**C**, **D**). **C** Near-infrared scanning laser ophthalmoscopy image with green arrow show the position of SD-OCT cross-section. D, SD-OCT image of the CRTs showing the irregular retinal region with hyporeflective cavities (open and black arrows). Vitreoretinal adhesion and traction sites (open arrowheads)

**Fig. 4:**
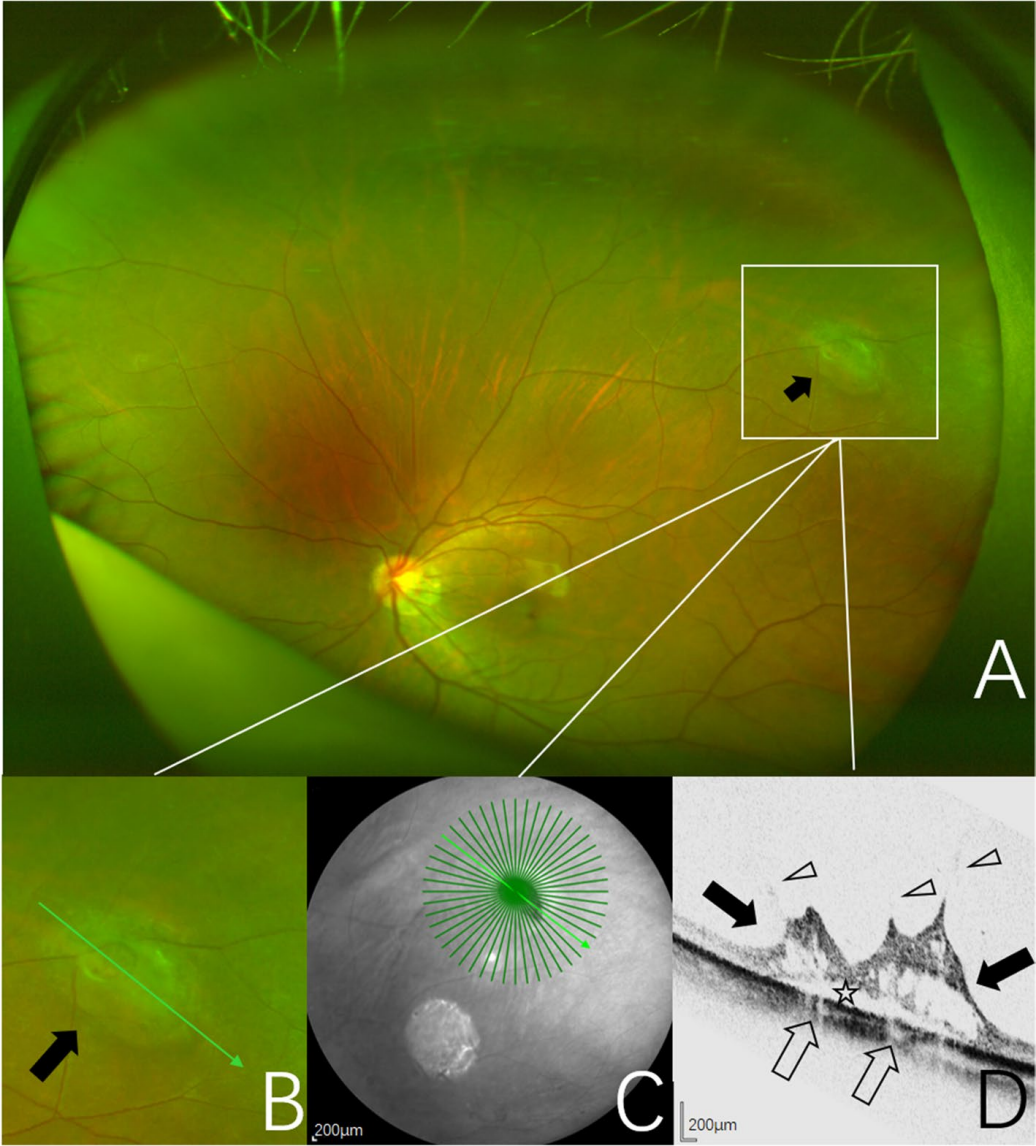
Images obtained from the right eye of a 63-year-old woman. **A** UWF pseudocolor image of one CRT (black arrow), outlined by the inset. **B** Pseudocolor image of magnification of CRT area outlined by the inset showing a large area of preretinal cotton-like structure with blurred borders and the retina in the center and at the edges of the lesion is gray and elevated (black arrow). The green arrow indicates the section captured by the SD-OCT in (**C**, **D**). **C** Near-infrared scanning laser ophthalmoscopy image with green arrow show the position of SD-OCT cross-section. **D** SD-OCT image of the CRTs showing an irregular retinal region with hyporeflective cavities (black arrows). Vitreoretinal adhesion and traction sites (open arrowheads). Shallow neuroepithelium detachment (open asterisk). Decreased choroidal reflectivity at the level of the retinal elevation (open arrows)

A total of 29 CRTs in the 26 eyes of 25 subjects were successfully imaged using SD-OCT with a 55° widefield lens. The mean age of the subjects was 38.6 years (range, 16–68 years; standard deviation, 18.3 years). On UWF images, the CRT lesion appeared as a focal, circular, cotton ball-like elevated structure frequently associated with vitreous traction. Nine cases (31.03%) were found pigmentation at the base. Topographically, all CRTs were found in the peripheral retina and were mainly distributed in the temporal quadrant of the peripheral retina. There were 12 cases (41.38%) in superotemporal quadrant, 12 cases (41.38%) in inferotemporal quadrant, 3 cases (10.34%) in superonasal quadrant and two case (6.90%) in inferonasal quadrant. On SD-OCT images (Figures 1, 2, 3 and 4), the CRTs exhibited hyperreflective irregular elevated lesions with internal hyporeflective cystoid cavities. Normal layers of the neuroepithelium could not be distinguished. The mean diameter of the long axis at the base of CRTs was 1022 microns (range, 117–3711 microns; standard deviation, 815 microns). There was condensed cortical vitreous adhesion and traction to the inner retina at the apex of the raised surface of all CRT lesions. Among them, retinal tears in 24.14% (7/29), suspected retinal tears in 27.59% (8/29), and shallow neuroepithelium detachment in 31.03% (9/29). The retinal pigment epithelium (RPE) was destructed and the normally hyperreflective RPE layer was focally absent. The elevated area of dense retina decreased choroidal reflectivity.

## Discussion

This study showed that the detailed anatomic information of CRT in the peripheral retina could be obtained by current clinical available 55° widefield SD-OCT devices. Taney et al. [6] revealed that the CRT as a focal, elevated gliotic lesion associated with vitreous traction in the peripheral retina by OCT imaging in vivo, but the intralesional cystic structure were not obvious on OCT images. In this study, the CRT on SD-OCT images exhibited hyperreflective irregular elevated lesions with internal hyporeflective cystoid cavities, and normal layers of the neuroepithelium could not be distinguished. Our results based on the widefield SD-OCT imaging were generally consistent with the characteristic intralesional cystic changes on histopathological analysis of CRT [2]. This demonstrates the utility of widefield SD-OCT imaging in detecting subtle features of the peripheral retinal lesions that are often hidden on standard ophthalmic examinations.

The CRT regions have a risk of progression toward vision-threatening retinal detachment because they may be avulsed by vitreous traction resulting in a full-thickness retinal break [2]. The CRT accounted for 7.2% ~ 16.1% in peripheral retinal alterations [7, 8]. Retinal tears at CRT regions may be responsible for as many as 10% of clinical retinal detachments associated with posterior vitreous detachment (PVD) [1]. Our study showed that vitreoretinal traction was found in all CRT regions, and retinal tears or suspected retinal tears were found in nearly half of cases. Suspicious retinal tear refers to CRT lesions that show poor continuity of the local retinal neuroepithelial layer in 55° widefield SD-OCT, but has not formed a clear full-thickness rupture, i.e. they haven’t met the standard diagnosis of typical retinal tear, and these CRT lesions are considered to be clinically without retinal tear. The reason may be that the resolution of the 55° widefield SD-OCT is not adequate to accurately distinguish all the fine structures of CRT lesions located in the peripheral retina, or that there is indeed a transitional stage between the very thin and complete rupture of retinal neuroepithelial layer, that is, a seemingly broken state. Asymptomatic and symptomatic retinal tears progress to rhegmatogenous retinal detachment in 0%-13.8% and 35%-47% of cases, respectively [9]. The treatment by prompt creation of a chorioretinal adhesion around these symptomatic tears can reduce the risk of retinal detachment to less than 5% [10]. While the need for prophylactic laser treatment for CRT remains controversial. A very early study reported the risk of CRT leading to retinal detachment was less than 1% (range, 0.18% to 0.28%), so prophylactic treatment of CRT was therefore not advised [2]. However, this result was based on the study of autopsy eyes and differed from the real-world data in vivo. There are likely more cases of CRTs involving retinal detachment that were not recognized because of the previous lack of availability of clinical high-resolution imaging technology in the peripheral retina. We observed that widefield SD-OCT imaging can accurately determine the presence of retinal tears at CRT regions. Therefore, prophylactic laser treatment should be considered for such cases of CRT with retinal tears and follow-up for those without such signs.

The technology of OCT has been steadily improved, and one of the advancements is the widening of the scanning angle. The conventional 30° SD-OCT scan modes have a limited scanning angle and hardly obtain clear images of the peripheral retina. Choudhry et al. [8] reported a novel technique to image the far retinal periphery using SD-OCT with a 30° steerable lens. Giannakaki et al. [11] demonstrated that the 55° widefield SD-OCT scans provided a good overview of the posterior pole and presented similar quantitative values as the standard 30°scan modes. ––It was previously discussed that morphological alterations of small lesions may be missed using 55° widefield imaging, for a lower horizontal resolution of 19.95 mm/pixel in the 55° widefield scans compared with 11.74 mm/pixel in the conventional 30° SD-OCT scans [12]. However, we only analyzed cross-sectional structural images of CRT in the peripheral retina using the 55° widefield SD-OCT imaging, so we cannot exclude that results may be different when evaluating CRT by the standard 30° OCT scan mode.

There are several limitations to this study and technique. First, the number of eyes analyzed remained relatively limited. Second, this technique is limited by poor dilation and media opacities. Third, there is no long-term follow-up of CRT.

## Conclusions

In summary, the 55° widefield SD-OCT imaging can provide detailed cross-sectional anatomic information of a subtle entity in periphery retina. This imaging technique may deepen our structural understanding of CRT and may influence decision-making in clinical practice.

## Data Availability

All data generated or analyzed during this study are included in this published article.
